# Melanoma Brain Metastases in the Era of Targeted Therapy and Checkpoint Inhibitor Therapy

**DOI:** 10.3390/cancers13071489

**Published:** 2021-03-24

**Authors:** John M. Rieth, Umang Swami, Sarah L. Mott, Mario Zanaty, Michael D. Henry, Aaron D. Bossler, Jeremy D. Greenlee, Yousef Zakharia, Marion Vanneste, Brooke Jennings, Mohammed M. Milhem

**Affiliations:** 1 Department of Internal Medicine, University of Iowa Hospitals and Clinics, Iowa City, IA 52242, USA; 2 Huntsman Cancer Institute, University of Utah Health, Salt Lake City, UT 84112, USA; umang.swami@hci.utah.edu; 3 Holden Comprehensive Cancer Center, University of Iowa Hospitals and Clinics, Iowa City, IA 52242, USA; sarah-mott@uiowa.edu (S.L.M.); yousef-zakharia@uiowa.edu (Y.Z.); mohammed-milhem@uiowa.edu (M.M.M.); 4 Department of Neurosurgery, University of Iowa Hospitals and Clinics, Iowa City, IA 52242, USA; mario-zanaty@uiowa.edu (M.Z.); jeremy-greenlee@uiowa.edu (J.D.G.); 5 Department of Molecular Physiology and Biophysics, Carver College of Medicine, University of Iowa, Iowa City, IA 52242, USA; michael-henry@uiowa.edu (M.D.H.); marion-vanneste@uiowa.edu (M.V.); brooke-jennings@uiowa.edu (B.J.); 6 Department of Pathology, Carver College of Medicine, University of Iowa, Iowa City, IA 52242, USA; aaron-bossler@uiowa.edu

**Keywords:** melanoma, prognosis, cancer management, clinical cancer research, metastasis, survival

## Abstract

**Simple Summary:**

Brain metastases are common in melanoma and are often associated with significant morbidity and mortality. Although many new treatments for melanoma have been approved in recent years, including immune checkpoint inhibitors and BRAF/MEK inhibitors, limited data are available for survival for patients with brain metastases treated with these novel therapies. The aim of this retrospective study was to evaluate current surgical, radiation, and systemic therapies over the past 10 years in melanoma patients with brain metastases. Our study noted increased overall survival in patients treated with craniotomy and CTLA-4 checkpoint inhibitors, while whole brain radiation was associated with poorer overall survival.

**Abstract:**

Brain metastases commonly develop in melanoma and are associated with poor overall survival of about five to nine months. Fortunately, new therapies, including immune checkpoint inhibitors and BRAF/MEK inhibitors, have been developed. The aim of this study was to identify outcomes of different treatment strategies in patients with melanoma brain metastases in the era of checkpoint inhibitors. Patients with brain metastases secondary to melanoma were identified at a single institution. Univariate and multivariable analyses were performed to identify baseline and treatment factors, which correlated with progression-free and overall survival. A total of 209 patients with melanoma brain metastases were identified. The median overall survival of the cohort was 5.3 months. On multivariable analysis, the presence of non-cranial metastatic disease, poor performance status (ECOG 2–4), whole-brain radiation therapy, and older age at diagnosis of brain metastasis were associated with poorer overall survival. Craniotomy (HR 0.66, 95% CI 0.45–0.97) and treatment with a CTLA-4 checkpoint inhibitor (HR 0.55, 95% CI 0.32–0.94) were the only interventions associated with improved overall survival. Further studies with novel agents are needed to extend lifespan in patients with brain metastases in melanoma.

## 1. Introduction

Of all malignancies, melanoma has the highest propensity to migrate to the brain, which is associated with a poor prognosis [1,2,3]. About 20–28% of patients have brain metastases at diagnosis [2,4], and about 49–75% of patients are found to have brain metastases on autopsy [5,6,7]. Additionally, the frequency of brain metastases in melanoma is thought to be on the rise due to the increased survival of patients diagnosed with melanoma. Previously, patients with melanoma had limited treatment options, including chemotherapy, whole-brain radiation therapy (WBRT), stereotactic radiosurgery (SRS), and surgical resection. Since 2011, checkpoint inhibitors and BRAF/MEK targeted therapy have revolutionized the treatment of melanoma, resulting in dramatically enhanced survival. Specifically, the first single-agent BRAF inhibitors were approved in 2011, single-agent ipilimumab in 2011, PD-1 inhibitors in 2014, and combination ipilimumab and nivolumab in 2015.

Despite recent advances in the treatment of advanced melanoma, brain metastases remain a significant source of morbidity and mortality, with a poor median overall survival. In a recent report, the weighted median overall survival with single-agent chemotherapy, immunotherapy alone, or targeted therapy were between five to nine months only [8]. Due to these dismal outcomes, continued study of brain metastases and treatments for brain metastases are needed.

**Figure 1:**
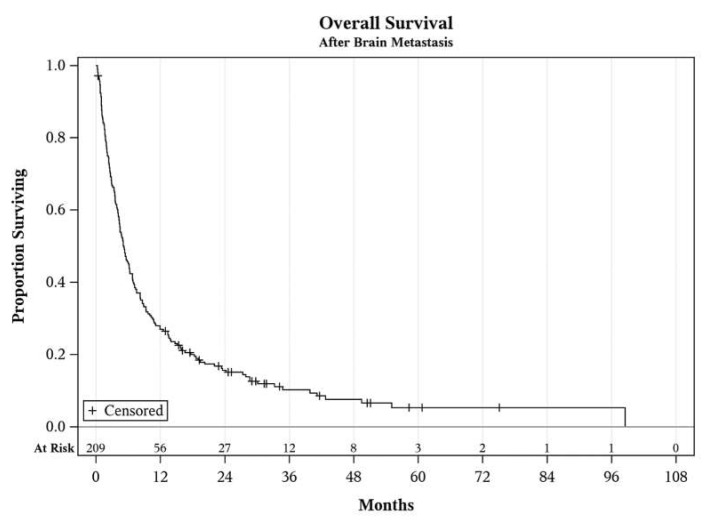
Overall the survival of the entire cohort after diagnosis of brain metastasis.

Many studies have suggested that novel therapies have efficacy against brain metastases in the clinical trial setting, including BRAF/MEK targeted therapy and immunotherapy [9,10,11,12,13]. These studies, however, do not address survival outcomes in patients who are not candidates for clinical trials. Additionally, few prospective clinical trials have compared different therapies for brain metastases in melanoma, making it difficult to assess different lines of immunotherapy and targeted therapies in this patient population. Here we aimed to study the outcomes of treatments for brain metastases by comparing different treatment strategies used during the immunotherapy era in the real-world setting (Figure 1).

## 2. Materials and Methods

After IRB approval, a total of 445 patients were identified with the assistance of TriNetX by searching EPIC at the University of Iowa, using the search terms “melanoma” and “dexamethasone.” All patient charts and imaging were manually reviewed, identifying 209 patients with melanoma brain metastases. Inclusion criteria included all identified patients treated with brain metastases at the University of Iowa from 8 January 2008 through 8 January 2019. Patients were excluded if treatment modalities used over the course of the disease could not be identified. Patient characteristics, including sex, ethnicity, histopathological subtype, location of primary, and BRAF status, were identified and recorded. All patients were staged according to the guidelines set by the AJCC 8th edition [14]. Dates of initial diagnosis, diagnosis of metastatic disease and identification of brain metastasis were also recorded. The number and locations of brain metastases were recorded, as well as the location of extracranial metastatic disease. Treatment modalities identified included systemic treatments (chemotherapy, checkpoint inhibitor therapy, targeted therapy), radiation therapy (SRS and WBRT), and surgical excision. Dates for all treatments were recorded, and indications for the cessation of systemic therapy were identified.

Cox regression models were used to assess the effects of clinical, pathological, and treatment variables on progression-free survival (PFS) and overall survival (OS). For PFS, time was calculated from diagnosis of brain metastasis to progression of brain metastasis by imaging Repsonce Evaluation Criteria in Solid Tumors RECIST 1.1 criteria [15], initiation of hospice services, loss to follow-up, or death due to any cause. For OS, time was calculated from brain metastasis to death due to any cause. Time-dependent covariates were incorporated to address concerns of immortal time bias by capturing changes in treatment (i.e., when each treatment was initiated) during the follow-up period after diagnosis of brain metastases. Estimated effects of predictors are reported as hazard ratios (HR) along with 95% confidence intervals. All statistical testing was two-sided and assessed for significance at the 5% level using SAS v9.4 (SAS Institute, Cary, NC, USA).

## 3. Results

### 3.1. Baseline Characteristics

A total of 209 patients with brain metastases secondary to melanoma were identified. The demographic and baseline characteristics at the time of diagnosis of brain metastasis are presented in Table 1. A majority (68.4%) of the patients were male. The median follow-up was 5.1 months (range 0.3–98.5). At the data cutoff, 21 patients were alive. Identification of a primary site and/or formal dermatology consultation for evaluation of a primary melanoma was conducted in 189 patients. The histological breakdown is described in Table 1. The cutaneous metastases (155 patients) are further characterized by site and histological subtype in Table 1. Twenty-six patients were designated as melanoma of unknown primary after a dermatology evaluation.

Upon diagnosis of brain metastasis, 108 (51.7%) had at least one metastasis greater than 2 cm in greatest diameter. The 87 patients with a single metastasis were categorized further, with locations of metastases described in Table 1. Of patients, who were diagnosed with melanoma prior to the discovery of brain metastases, the median time from diagnosis of brain metastases to brain metastasis discovery was 38.7 months.

### 3.2. Treatment

Of all the patients diagnosed with brain metastasis, 57 patients were treated with adjuvant therapy, which included—interferon (21 patients), GM-CSF (16 patients), PD-1 inhibitors (7 patients), CTLA-4 inhibitors (6 patients), seviprotimut-L (5 patients) and talimogene laherparepvec, transgenic lymphocytes/interferon, dabrafenib/trametinib, and a combination of IL-2, cisplatin, vinblastine, and dacarbazine (1 patient each).

A total of 82 patients (including 31 previously treated with adjuvant therapy) were treated for metastatic melanoma before the discovery of brain metastases. Of these patients, 48 had one line of therapy, 19 had two, 11 had three, three had five, two had four, and one patient had six. Of these patients, 25 were exposed to ipilimumab before the development of brain metastases, 29 were exposed to a PD-1 checkpoint inhibitor, and 25 were treated with a BRAF ± MEK inhibitor.

Most patients (133) were treated with systemic therapy after the diagnosis of brain metastases. First-line therapies consisted of PD-1 inhibitors (45 patients), cytotoxic chemotherapy (37 patients), BRAF/MEK inhibitors (22 patients), ipilimumab (18 patients), a combination of ipilimumab with nivolumab (3 patients), and other therapies (8 patients). Of the treated patients, 77 were treated with a single line of therapy, 36 had two lines of therapy, 13 had three lines of therapy, and 7 had four lines of therapy. Initial salvage therapies after progression included PD-1 and/or CTLA-4 checkpoint inhibitors in 22 patients, BRAF/MEK inhibitors in 13, chemotherapy in 17, and other therapies in 4. In total, 63 patients were eventually treated with a PD-1 inhibitor after diagnosis of brain metastasis, 41 with BRAF/MEK inhibitor, and 24 with ipilimumab.

In total, 74 patients (35.4%) underwent craniotomy with tumor resection, of which 64 patients had the tumor resection within one month of diagnosis of brain metastasis. Patients were selected for surgery based on evaluation and discussion by neurosurgeons and radiation oncologists. Two separate patients received a brain biopsy for diagnosis, and an additional two patients underwent ventriculostomy for ventricular obstruction. Surgical resections were performed on 50 patients with a single metastasis, nine with two metastases, four with three metastases, eight with four metastases, and three with five or greater metastasis. Of patients, who underwent craniotomy, 64 (86.5% of all patients with surgical resection) had a primary brain lesion greater than 2 cm in diameter. Of the 66 patients, of which operative reports were able to be obtained, 63 tumors were noted to be gross total resections.

Regarding radiation therapy, 116 had SRS, including all but one of the patients treated with craniotomy. WBRT was used for 107 patients, including 44 patients who underwent both SRS and WBRT.

### 3.3. Outcomes

The median OS survival of the entire cohort was 5.2 months (see Figure 1.). Concerning initial treatments after diagnosis of brain metastasis, median intracranial PFS with immune checkpoint inhibitors (PD-1 or CTLA-4 inhibitors as a single agent or in combination) was 6.3 months. The median PFS after initial management with BRAF/MEK inhibitors was 5.3 months, and after cytotoxic chemotherapy was only 3.6 months. Intracranial progression after systemic treatment was determined by patient death in 35 patients, development of new brain metastases in 53 patients, development of leptomeningeal involvement in 5 patients, and increase in intracranial tumor size without new metastasis in 28 patients. A total of 11 patients did not have a progression of their metastases and were alive as of the analysis.

Of the 74 craniotomy patients, 14 died prior to brain imaging surveillance after craniotomy. A total of 23 patients had stable surveillance imaging, of which 12 were still alive as of this analysis. The remaining 37 patients had intracranial progression after craniotomy with a median time to progression of 119 days and, of which 2 were alive as of the time of analysis. Progression was secondary to new intracranial metastases in 21 patients, development of leptomeningeal disease in 3 patients, and local recurrence in 7; progression was otherwise due to progression of unresected lesions.

In the 107 patients treated with WBRT, 35 died prior to surveillance imaging. A total of 16 had stable disease, of which 4 were alive at the time of analysis. The remaining 56 had intracranial progression, with a median time to progression of 59.5 days. In 25 patients, progression was determined by new metastases, and development of leptomeningeal involvement in 4 patients, while the remainder was noted to have increased size of previously noted lesions only.

Of the 116 patients treated with SRS, 13 died prior to additional imaging. A further 26 patients had stable disease, with 13 still alive as of the time of analysis. A total of 77 progressed, with a median time to progression of 103 days. New metastases were identified in 60 patients on the first progression after SRS, and 4 developed the leptomeningeal disease, while the remainder only had local progression.

**Figure 2:**
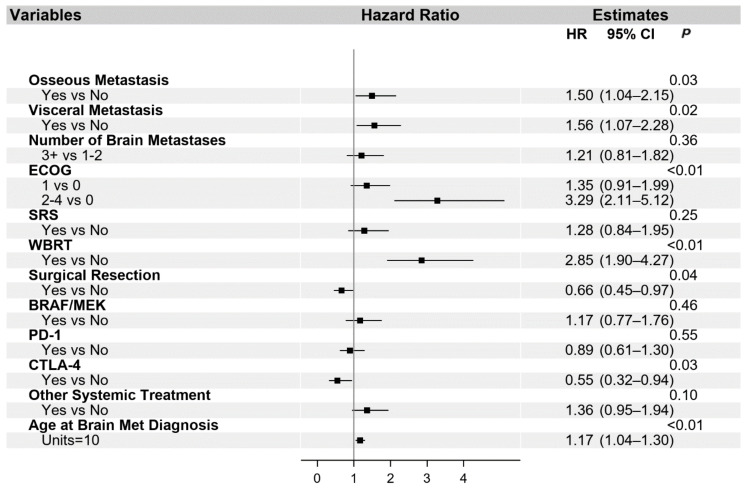
Multivariable analysis of overall survival. Abbreviated variable names include Eastern Cooperative Oncology Group performance status (ECOG), stereotactic radiosurgery (SRS), whole-brain radiation therapy (WBRT), and metastasis (met).

**Figure 3:**
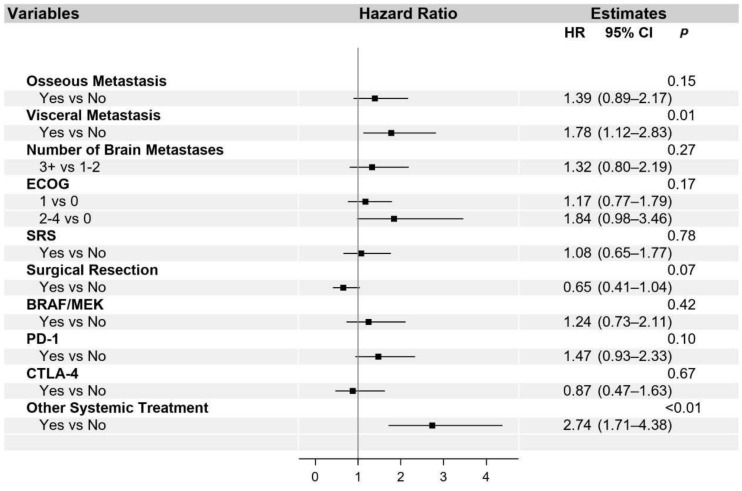
Multivariable analysis of progression-free survival. Abbreviated variable names include Eastern Cooperative Oncology Group performance status (ECOG), stereotactic radiosurgery (SRS), whole-brain radiation therapy (WBRT), and metastasis (met).

The univariate and multivariable analysis for OS is presented in Table S1 and in Figure 2, respectively. Additionally, univariate (Table S2) and multivariable (Figure 3) analyses were performed for the PFS of systemic interventions. On multivariable analysis, the presence of non-cranial metastatic disease, poor performance status (ECOG 2–4), WBRT, and older age at diagnosis of brain metastasis was associated with poorer OS. WBRT, in particular, was associated with a hazard ratio of 2.85 (95% CI 1.90–4.27). Craniotomy (HR 0.66, 95% CI 0.45–0.97) and treatment with a CTLA-4 checkpoint inhibitor (HR 0.55, 95% CI 0.32–0.94) were the only interventions associated with improved OS. The overall reduced risk of death in patients treated with craniotomy was 34%, and in patients treated with CTLA-4, checkpoint inhibition was 45%. With regard to PFS, visceral metastasis and non-checkpoint and BRAF directed therapies were associated with poorer outcomes.

PD-1 and CTLA-4 checkpoint inhibitors were evaluated separately in the above analysis. Assessment of a synergistic effect of the PD-1 and CLTA-4 inhibitors, when administered in combination, was not found to be statistically significant, which may be a result of a small sample size.

## 4. Discussion

Here we present a cohort of 209 melanoma patients with intracranial metastases. Historically, patients with brain metastases have had a very poor prognosis, with previous retrospective studies identifying as few as 2.4% surviving greater than three years, with the cause of death in 94.5% attributable to brain metastases [16].

Our study confirms the findings of previous epidemiological studies remarking on the higher prevalence of brain metastases among male patients. Interestingly, a surprisingly high percentage (12.4%) of patients had melanoma of unknown primary even after excluding the patients who never had a dermatology evaluation. A retrospective review in 2017 by Utter et al. on melanomas of unknown primaries found that 79% of patients with melanoma of unknown primary developed brain metastases [17]. It is possible that the biology of melanomas of unknown primary may uniquely predispose these patients to brain metastases. Alternatively, differences in the host immune response to melanomas at different organ sites might shape this behavior.

In this series, ipilimumab was found to be associated with improved OS, while PD-1 checkpoint inhibitors were not associated with improved outcomes in the multivariable analysis and were associated with poorer PFS. Recent prospective clinical trials by Long et al. [18] and Tawbi et al. [13] treated patients with combined CTLA-4 and PD-1 checkpoint inhibition of brain metastasis, with evidence of clinical benefit in a substantial portion of patients with brain metastases. Silk et al. also found the efficacy of monotherapy ipilimumab in brain metastases due to melanoma when combined with radiation therapy [19]. In contrast with our data, a recent retrospective study of 79 patients with melanoma and brain metastases by Vosoughi et al. found that treatment with a PD-1 checkpoint inhibitor had enhanced median OS of 37.9 months as compared to the 12.8 month median OS of the whole cohort [8]. Prospective trials, including treatment with pembrolizumab by Kluger et al. and Goldberg et al. in addition to a prospective trial involving treatment with nivolumab by Long et al., have also demonstrated activity against brain metastases, with median overall survival ranging from 8.7 to 17 months [18,20,21]. The study by Long et al. [18] compared ipilimumab and nivolumab-to-nivolumab monotherapy, but no statistically significant differences could be identified due to sample size. Additional studies, including PD-1 checkpoint inhibitor monotherapy have had mixed results, with a less optimistic median OS from 9.9 to 18.5 months [22,23,24,25]. The rationale for the efficacy of ipilimumab compared with the PD-1 inhibitors is unclear but possibly reflects the unique microenvironment and immunology of the brain.

Treatment with ipilimumab was also found to be associated with better outcomes than BRAF/MEK inhibitors. Prospective studies by Dummer et al., McArthur et al., and Long et al. have found the response of melanoma brain metastases to single-agent BRAF therapy, although median progression-free survival and median overall survival were short [9,10,26]. Similarly, BRAF/MEK combination therapy was noted to have some responses in the prospective clinical trial by Davies et al., but the length of response to combined therapy was short, with a median progression-free survival of 5.6 months and a median overall survival of 10.8 months [11]. Unfortunately, few prospective clinical trials have compared different therapies for brain metastases in melanoma, making it difficult to contrast different lines of immunotherapy and targeted therapies in this patient population.

Additionally, our study confirms that craniotomy with surgical resection resulted in better outcomes, which is consistent with previous studies [27]. However, other confounding variables could have contributed to this result, including the selection criteria for craniotomy not accounted for by the ECOG. Of note, 50% of patients treated with craniotomy had a recurrence of intracranial disease after surgical resection, with a majority (56.8%) progressing due to the development of new brain metastases. Others have reported a recurrence rate as high as 48–55% after surgical resection, which is similar to our observations [28].

Although SRS resulted in better outcomes in the univariate analysis, this survival advantage was not found in the multivariable analysis. In our study, WBRT was associated with decreased OS. This is in agreement with current National Comprehensive Cancer Network guidelines, which do not recommend upfront WBRT for melanoma patients with brain metastasis [29]. Moreover, a randomized phase 3 study that enrolled patients with brain metastasis due to melanoma and other tumor types also has demonstrated a worse cognitive decline without improvement in overall survival with WBRT as compared to SRS [30].

One limitation of this study is the retrospective design, which is common to other retrospective studies, including the risk of confounding bias and inability to determine causation. Adjustment for potential confounders, such as ECOG and age, in the multivariable analyses, was done. However, there may be additional confounding factors not captured in the study, which may limit interpretations. These findings should be further validated by additional external cohorts and prospective clinical trials.

## 5. Conclusions

This study reported on the baseline characteristics and impact of treatment on melanoma brain metastases. Importantly, multivariable analysis systemic treatment with ipilimumab was associated with better OS, while WBRT was associated with worse OS. However, even with these promising results with ipilimumab, OS remains poor. Further studies with novel agents and combinations are warranted to identify efficacious treatment for brain metastases in melanoma.

## Figures and Tables

**Table 1:** Baseline characteristics of patients with brain metastases.

Characteristic	Variable	Sub-Variable	*n* = 209	Percent
Sex				
-	Male	-	143	68.4%
-	Female	-	66	31.6%
Survival				
-	Living	-	21	10.0%
-	Deceased		188	90.0%
Location of Primary		-	-	-
-	Cutaneous		155	82.0%
-	Mucosal	-	4	2.1%
-	Uveal	-	3	1.6%
-	Primary CNS		1	0.5%
-	Melanoma of unknown primary		26	13.8%
-	No dermatology confirmed lesion		20	-
Initial stage				
-	IA	-	12	5.9%
-	IB	-	20	9.9%
-	IIA	-	17	8.4%
-	IIB	-	10	5.0%
-	IIC	-	10	5.0%
-	IIIA	-	9	4.5%
-	IIIB	-	20	9.9%
-	IIIC	-	28	13.9%
-	IIID	-	3	1.5%
-	IV	-	58	28.7%
-	Multiple primaries		15	7.4%
-	Undocumented		7	-
BRAF status				
-	BRAF mutation		87	60.8%
-	BRAF wild-type		56	39.1%
-	Missing	-	66	-
Brain metastasis as the presenting symptom				
-	Yes	-	41	19.6%
-	No	-	168	80.4%
Extracranial metastatic disease on the diagnosis of brain metastasis				
-	Yes	-	179	85.6%
-		Lung	127	60.8%
-		Liver	59	28.2%
-		Osseous	52	24.9%
-		Mesentery/GI tract	38	18.2%
-		Adrenal	34	16.3%
-		Spleen	17	8.1%
-		Leptomeninges	5	2.4%
-	No	-	30	14.4%
ECOG at diagnosis of brain metastasis				
-	0	-	66	31.6%
-	1	-	87	41.6%
-	2	-	39	18.7%
-	3	-	15	7.2%
-	4	-	2	1.0%
Number of brain metastases on initial diagnosis				
-	1	-	87	41.6%
-	2	-	24	11.5%
-	3	-	18	8.6%
-	4	-	22	10.5%
-	>5	-	58	27.8%
Initial size of largest intracranial metastasis				
-	<2	-	101	48.3%
-	>2	-	108	51.7%
Characteristics of cutaneous melanoma				
Characteristic	Variable	-	*n* = 155	Percent
Location				
-	Torso	-	72	46.5%
-	Head and neck		39	25.2%
-	Upper extremity		15	9.7%
-	Lower extremity		15	9.7%
-	Multiple primaries		14	9.0%
Histological subtype				
-	Superficial spreading		50	39.1%
-	Nodular	-	41	32.0%
-	Lentigo maligna melanoma		10	7.8%
-	Desmoplastic		4	3.1%
-	Acral lentiginous		3	2.3%
-	Polyploid	-	2	1.6%
-	Regressed		2	1.6%
-	Spitz nevus		1	0.8%
-	Blue nevus		1	0.8%
-	Multiple primaries		14	10.9%
-	Undocumented		27	-
Characteristics of single brain metastases				
Characteristic	Variable	-	*n* = 87	Percent
-	Frontal lobe	-	31	35.6%
-	Temporal lobe		15	17.2%
-	Parietal lobe		13	14.9%
-	Occipital lobe		10	11.5%
-	Cerebellum		10	11.5%
-	Basal ganglia		3	3.4%
-	Brain stem		1	1.1%
-	Insula		2	2.3%
-	Pineal gland		1	1.1%
-	Ventricle		1	1.1%

## Data Availability

Further data available on request from the corresponding author. The data are not publicly available due to patient privacy.

## Supplementary Materials

The following are available online at https://www.mdpi.com/2072-6694/13/7/1489/s1, Table S1: Univariate and multivariable analysis for OS, Table S2: Univariate analysis for PFS of systemic interventions.
