# Supplementary material for: Melanoma Brain Metastases in the Era of Targeted Therapy and Checkpoint Inhibitor Therapy
Source: Cancers (Basel). 2021 Mar 24;13(7):1489. doi: 10.3390/cancers13071489 (PMC8037963; doi:10.3390/cancers13071489)
Supplement: Supplementary file 1 [file cancers-13-01489-s001.pdf]

cancers

MDPI

Supplementary Material: Melanoma Brain Metastases in the Era of Targeted Therapy and Checkpoint Inhibitor Therapy

John M. Rieth, Umang Swam, Sarah L. Mott, Mario Zanaty, Michael D. Henry, Aaron D. Bossler, Jeremy D. Greenlee, Yousef Zakharia, Marion Vanneste, Brooke Jennings and Mohammed M. Milhem

Table S1. Univariate and multivariable analysis for OS.

		Overall Survival			
**Variable**	**Comparison**	**Hazard Ratio**	**95% CI**		***P*-value**
Sex	Male vs Female	1.09	0.80	1.49	0.57
Brain met on diagnosis?	Yes vs No	1.04	0.73	1.49	0.81
Number of brain mets	3+ vs 1-2	2.11	1.57	2.82	**<0.01**
ECOG	1 vs 0	1.61	1.13	2.29	**<0.01**
	2-4 vs 0	4.15	2.79	6.18	-
Therapy prior to brain met?	Yes vs No	0.84	0.63	1.13	0.25
Initial Size of Largest Met (cm)	2+ vs <2	1.05	0.79	1.40	0.73
Metastatic Disease at BM diagnosis	Yes vs No	2.53	1.58	4.05	**<0.01**
Osseous Metastasis	Yes vs No	1.58	1.14	2.19	**<0.01**
Visceral Metastasis	Yes vs No	1.91	1.35	2.71	**<0.01**
SRS	Yes vs No	0.52	0.38	0.71	**<0.01**
Whole Brain	Yes vs No	2.87	2.13	3.87	**<0.01**
Surgery	Yes vs No	0.55	0.40	0.75	**<0.01**
BRAF/MEK	Yes vs No	1.30	0.90	1.88	0.16
PD-1	Yes vs No	0.72	0.51	1.01	0.06
CTLA4	Yes vs No	0.59	0.37	0.94	**0.03**
Other (Chemo + Epigenetic + Other)	Yes vs No	1.71	1.25	2.34	**<0.01**
Year of Brain Met	Units = 1	0.96	0.93	1.00	0.07
Age at Brain Met	Units = 10	1.10	1.00	1.21	0.05

Table S2. Univariate analysis for PFS of systemic interventions.

		Progression-Free Survival			
**Variable**	**Comparison**	**Hazard Ratio**	**95% CI**		***P*-value**
Sex	Male vs Female	1.16	0.79	1.7	0.45
Brain met on diagnosis?	Yes vs No	1.48	0.94	2.32	0.09
Number of brain mets	3+ vs 1-2	1.64	1.14	2.37	<0.01
ECOG	1 vs 0	1.38	0.94	2.03	**0.04**
	2-4 vs 0	2.05	1.16	3.64	-
Therapy prior to brain met?	Yes vs No	0.71	0.50	1.02	0.07
Initial Size of Largest Met (cm)	2+ vs <2	0.84	0.59	1.21	0.36
Metastatic Disease at BM diagnosis	Yes vs No	2.60	1.50	4.53	**<0.01**

		Progression-Free Survival			
**Variable**	**Comparison**	**Hazard Ratio**	**95% CI**		***P*-value**
Osseous Metastasis	Yes vs No	1.75	1.16	2.63	**<0.01**
Visceral Metastasis	Yes vs No	1.97	1.30	2.98	**<0.01**
SRS	Yes vs No	0.67	0.46	0.98	**0.04**
Surgery	Yes vs No	0.55	0.38	0.81	**<0.01**
BRAF/MEK	Yes vs No	1.37	0.89	2.11	0.15
PD-1	Yes vs No	0.90	0.61	1.31	0.57
CTLA4	Yes vs No	0.76	0.47	1.25	0.29
Other (Chemo + Epigenetic + Other)	Yes vs No	2.55	1.77	3.68	<0.01
Year of Brain Met	Units=1	0.96	0.92	1.01	0.10
Age at Brain Met	Units=10	1.04	0.92	1.17	0.53

Publisher's Note: MDPI stays neutral with regard to jurisdictional claims in published maps and institutional affiliations.

© 2021 by the authors. Licensee MDPI, Basel, Switzerland. This article is an open access article distributed under the terms and conditions of the Creative Commons Attribution (CC BY) license (http://creativecommons.org/licenses/by/4.0/).
